# Webster’s Unabridged

**Published:** 1868-11

**Authors:** 


### WEBSTER'S UNABRIDGED—ILLUSTRATED.

In all the essential points of a good dictionary, in the amplitude and selectness of its vocabulary, in the fullness and perspicacity of its definitions, in its orthœpy and (*cum grano salis*) its orthography, in its new and trustworthy etymologies, in the elaborate, but not too learned treatises, of its Introduction, in its carefully prepared and valuable appendices,—briefly, in its general accuracy, completeness, and practical utility,—the work is one which *none who read or write can henceforward afford to dispense with.*

